# Lower bicarbonate level is associated with CKD progression and all-cause mortality: a propensity score matching analysis

**DOI:** 10.1186/s12882-022-02712-y

**Published:** 2022-03-04

**Authors:** Hirotaka Fukasawa, Mai Kaneko, Yuri Uchiyama, Hideo Yasuda, Ryuichi Furuya

**Affiliations:** 1 Renal Division, Department of Internal Medicine, Iwata City Hospital, 512-3 Ohkubo, Iwata, Shizuoka 438-8550 Japan; 2 First Department of Medicine, Hamamatsu University School of Medicine, Hamamatsu, Shizuoka Japan

**Keywords:** Chronic kidney disease, Creatinine, End-stage kidney disease, Mortality, Serum bicarbonate level

## Abstract

**Background:**

Although metabolic acidosis is known as a potential complication of chronic kidney disease (CKD), there is limited information concerning the association between metabolic acidosis and clinical outcomes.

**Methods:**

Five hundred fifty-two patients referred to renal division of Iwata City Hospital from 2015 to 2017 were included as a retrospective CKD cohort, and finally 178 patients with CKD stage III or IV and 20 to 80 years of age were analyzed. We examined the association between serum bicarbonate (HCO_3_^−^) levels and clinical outcomes using Kaplan-Meier methods after the matching of baseline characteristics by propensity scores.

**Results:**

Of 178 patients with CKD, patients with lower HCO_3_^−^ levels (*N* = 94), as compared with patients with higher HCO_3_^−^ levels (*N* = 84), were more likely to be male (*P* < 0.05), had more severe CKD stages (*P* < 0.05), more frequent use of renin-angiotensin system inhibitor (*P* < 0.05) or uric acid lowering agent (*P* < 0.001), heavier body weight (*P* < 0.001) and lower estimated glomerular filtration rate (*P* < 0.05). In Kaplan-Meier analysis after propensity score matching, the incidence of composite outcome as the doubling of serum creatinine level from baseline, end-stage kidney disease requiring the initiation of dialysis, or death from any causes was significantly fewer in the higher HCO_3_^−^ group than the lower HCO_3_^−^ group (*N* = 57 each group, *P* = 0.016).

**Conclusions:**

Lower HCO_3_^−^ level is significantly associated with the doubling of serum creatinine level, end-stage kidney disease or all-cause mortality in patients with CKD.

**Trial registration:**

This study was registered with the Clinical Trial Registry of the University Hospital Medical Information Network (http://www.umin.ac.jp/, study number: UMIN000044861).

## Background

Metabolic acidosis is one of the first recognized complications in patients with chronic kidney disease (CKD) [1–3]. In patients with CKD, there is a direct correlation between the decline of estimated glomerular filtration rate (eGFR) and the reduction in serum bicarbonate (HCO_3_^−^) levels, which is thought to be caused by the inability of the kidney to synthesize ammonia, regenerate HCO_3_^−^, and excrete hydrogen ions (H^+^) [4].

Observational studies have shown an independent association between serum HCO_3_^−^ levels and adverse kidney outcomes [5–7], and small interventional trials of alkali supplementation or dietary modification have reported potential benefits of raising serum HCO_3_^−^ levels in patients with CKD [8–10]. On the other hand, there is limited information concerning the significant association between metabolic acidosis and clinical outcomes.

In this study, we examined the effects of metabolic acidosis on kidney disease progression and mortality in patients with CKD using the method of propensity score matching analyses.

## Materials and methods

### Study design

We conducted a retrospective observational cohort study. We included all patients referred to Iwata City Hospital (Shizuoka, Japan) from January 1, 2015 to December 31, 2017. Eligible patients were adults (more than 20 years of age), although patients over 80 years of age were excluded due to their life expectancy.

This study was approved by the institutional ethics committee (approval number: 2021–020) and conducted in accordance with the Declaration of Helsinki. Because of its retrospective nature, informed consent was waived. This study was registered with the Clinical Trial Registry of the University Hospital Medical Information Network (http://www.umin.ac.jp/, study number: UMIN000044861).

### Data collection and parameters analyzed

Clinical information including comorbidities and laboratory findings was collected by the access to patient’s records. Complete blood count was measured using XN-3000™ hematology analyzer (Sysmex Corporation, Hyogo, Japan) and serum electrolytes, albumin, low-density lipoprotein (LDL) cholesterol, creatinine, and C-reactive protein (CRP) levels were measured using Ci16200 auto-analyzer (Canon Medical Systems, Tochigi, Japan). Serum HCO_3_^−^ levels were measured using ABL90 FLEX blood gas analyzer (Radiometer medical ApS, Denmark). Normal range of serum HCO_3_^−^ levels were generally from 22 to 29 mEq/L [11, 12].

The demographic characteristics included age, sex, chronic kidney disease (CKD) stage, original kidney disease, the history of cardiovascular disease (CVD), medication, systolic blood pressure and body weight. Blood parameters included hemoglobin, albumin, LDL cholesterol, CRP, estimated glomerular filtration (eGFR), albumin-corrected calcium (Ca)-phosphorus (Pi) product and serum HCO_3_^−^ level. Urine parameters included spot urine proteinuria score by dipstick (coded as four grades of 0 to 4 according to 0, 1+, 2+, 3+, and 4+ and as 0.5 if ±).

### Outcome

The all patients were followed until January 1, 2020 or the incidence of the composite outcome, whichever occurred first. The outcome, assessed in a time-to-event analysis, was a composite of the doubling of serum Cr level from baseline, end-stage kidney disease requiring the initiation of dialysis, or death from any causes.

### Statistical analyses

Data were expressed as the mean ± standard deviation (SD) for continuous variables with normal distributions or the median and interquartile range (25th to 75th percentiles) for data with skewed distributions. The threshold for statistical significance was set at *P* < 0.05. Comparisons between two groups were performed using the Mann-Whitney *U*-test for continuous variables or the chi-squared test for categorical variables.

To adjust the differences in the baseline characteristics and perform the following analyses, propensity score matching was employed [13, 14]. To calculate the propensity score, the target threshold of serum HCO_3_^−^ level was set at 25.3 mEq/L based on its median value. The probability to over this threshold, as the propensity score, was determined by a multivariate logistic regression using the aforementioned 16 baseline characteristics including age, sex, CKD stage, original kidney disease, the history of CVD, medication, systolic blood pressure, body weight, hemoglobin, albumin, LDL cholesterol, CRP, eGFR, albumin-corrected Ca-Pi product and urinary protein score. A nearest-neighbor matching algorithm was used to match patients within a caliper width of 0.03 of the logit of the propensity scores. Outcome curves were constructed with Kaplan-Meier method and compared with the log-rank test. For Kaplan-Meier method, we analyzed the composite outcome as the doubling of serum Cr level from baseline, end-stage kidney disease requiring the initiation of dialysis, or death from any causes by the time to the first event. All statistical analyses were performed using IBM SPSS statistical software, version 26.0 (IBM SPSS, Tokyo, Japan).

## Results

### Patients

**Fig. 1:**
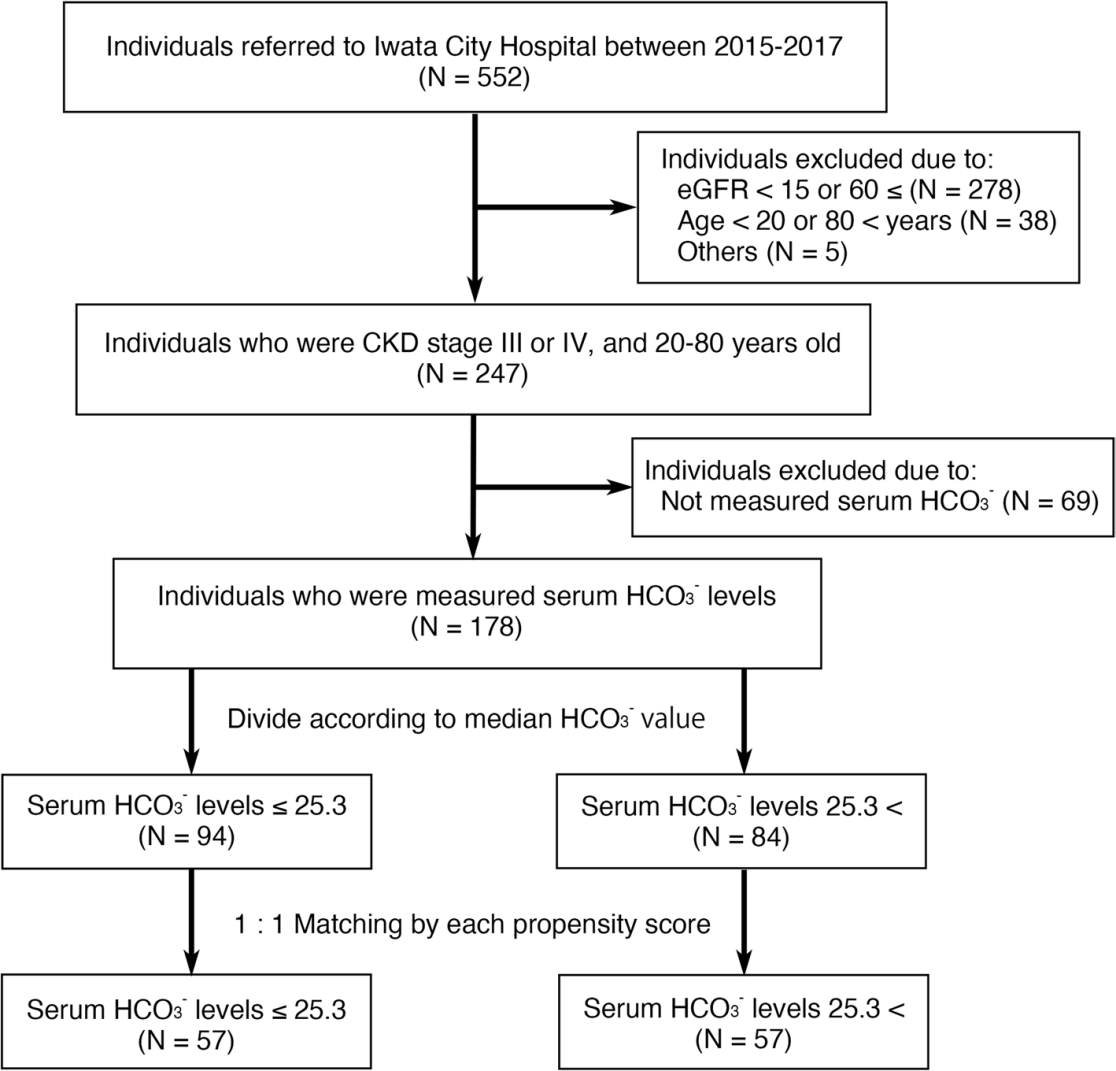
Study flow chart. Abbreviation: CKD, chronic kidney disease

During 3 years from January 1, 2015 to December 31, 2017, 552 patients were referred to renal division of Iwata City Hospital for the evaluation of proteinuria, hematuria or renal insufficiency. Among 552 patients, 247 patients were CKD stage III or IV and 20 to 80 years of age, although 69 patients were not measured serum HCO_3_^−^ levels. Finally, 178 patients were included in the following analyses (Fig. 1).

### Patient’s characteristics

In 178 patients analyzed, the mean age was 65 ± 10 years old and 104 patients (58.4%) were male. The original diseases were as follows: overt diabetic nephropathy in 18 patients (10.1%), chronic glomerulonephritis in 55 patients (30.9%), nephrosclerosis in 81 patients (45.5%), and polycystic kidney disease in 3 patients (1.7%). The mean eGFR was 41 ± 13 mL/min/1.73 m^2^ and the median value of serum HCO_3_^−^ levels was 25.3 mEq/L (Table 1).

**Table 1:**
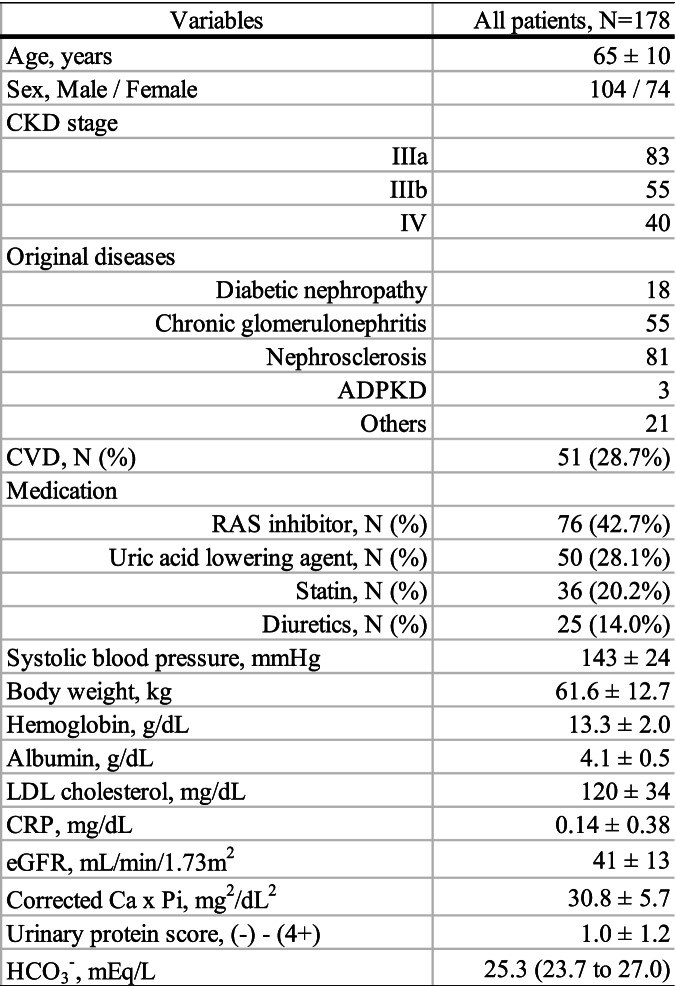
Patient characteristics

All variables are expressed as the mean ± SD or the median and interquartile range (25th to 75th percentiles)

Abbreviations: *ADPKD* autosomal dominant polycystic kidney disease; *Ca* calcium; *CKD* chronic kidney disease; *CRP* C-reactive protein; *CVD* cardiovascular disease; *eGFR* estimated glomerular filtration rate; *HCO*_*3*_^*−*^ bicarbonate; *LDL* low-density lipoprotein; *pi* inorganic phosphorus; *RAS* renin-angiotensin system; *SD* standard deviation

Next, we divided those 178 patients into lower and higher HCO_3_^−^ groups according to the median value of serum HCO_3_^−^ levels (*N* = 94 and 84, respectively, Table 2). Patients with lower HCO_3_^−^ levels, as compared with patients with higher HCO_3_^−^ levels, were more likely to be male (*P* < 0.05), had more severe CKD stages (*P* < 0.05), more frequent use of renin-angiotensin system (RAS) inhibitor (*P* < 0.05) or uric acid lowering agent (*P* < 0.001), heavier body weight (*P* < 0.001) and lower eGFR (*P* < 0.05).

**Table 2:**
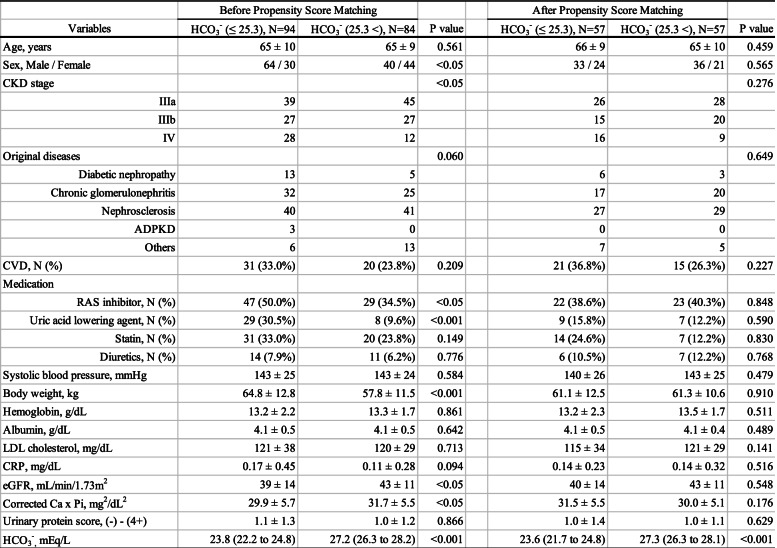
Patient characteristics of lower (serum HCO_3_^−^ levels ≤25.3 mEq/L) and higher HCO_3_^−^ groups (25.3 < mEq/L) before and after propensity score matching

All variables are expressed as the mean ± SD or the median and interquartile range (25th to 75th percentiles)

Comparisons between lower and higher HCO_3_^−^ groups were performed using the Mann-Whitney *U* test or chi-square test

Abbreviations: *ADPKD* autosomal dominant polycystic kidney disease; *Ca* calcium; *CKD* chronic kidney disease; *CRP* C-reactive protein; *CVD* cardiovascular disease; *eGFR* estimated glomerular filtration rate; *HCO*_*3*_^*−*^ bicarbonate; *LDL* low-density lipoprotein; *pi* inorganic phosphorus; *RAS* renin-angiotensin system; *SD* standard deviation

### Adjustment of patient’s characteristics with propensity score matching

Next, to compare the clinical outcomes between lower and higher HCO_3_^−^ groups under the similar background, we employed the method of propensity score matching. After the propensity score matching, the differences of patient’s characteristics at the baseline between lower and higher HCO_3_^−^ groups were adjusted (*N* = 57 each group, Table 2).

### Outcome

**Fig. 2:**
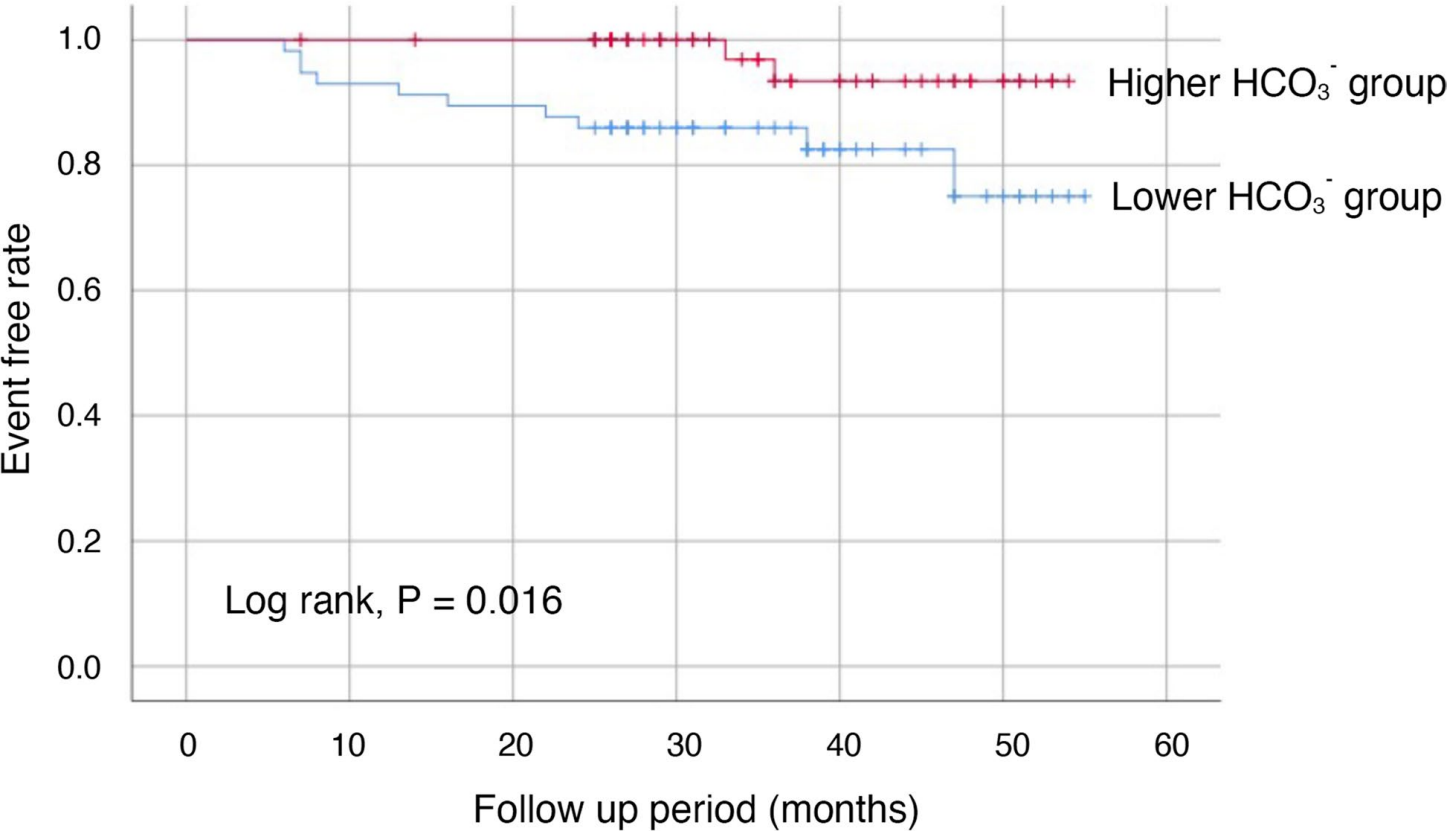
Kaplan-Meier curves of lower (serum HCO_3_^−^ levels ≤25.3 mEq/L) and higher HCO_3_^−^ groups (25.3 < mEq/L) after propensity score matching

The all patients were followed until January 1, 2020 and were evaluated the incidence of clinical outcomes. The mean follow-up period was 36 ± 12 months. In 114 patients after propensity score matching (*N* = 57 each group), the incidence of composite outcomes as the doubling of serum Cr level from baseline, end-stage kidney disease requiring the initiation of dialysis, or death from any causes was significantly less in the higher HCO_3_^−^ group than the lower HCO_3_^−^ group, occurring in 2 patients (3.5%) and 10 patients (17.5%), respectively (*P* = 0.016, Fig. 2).

## Discussion

In the present study, we showed that lower HCO_3_^−^ level was significantly associated with the incidence of composite outcomes as the doubling of serum creatinine level, end-stage kidney disease requiring the initiation of dialysis and all-cause mortality in patients with CKD, after the matching for baseline characteristics and laboratory data using propensity scores.

Experimental studies in animals have showed that metabolic acidosis contributes to progression of CKD [15, 16], and observational studies in humans have found the association between metabolic acidosis and worsening kidney function [5–7]. A small number of interventional trials have also showed that sodium bicarbonate (NaHCO_3_) supplementation can slow the rate of kidney disease progression [9, 17].

Several mechanisms how metabolic acidosis accelerates the progression of CKD are proposed. Nath et al. [18] have reported that metabolic acidosis stimulates ammonia production that increases acid excretion, but leads to ammonia-induced complement activation and deposition of C3 and C5b-9 that can cause tubulo-interstitial injury. Wesson et al. [19, 20] have showed that metabolic acidosis in CKD stimulates the production of intra-kidney paracrine hormones including angiotensin II, aldosterone, and endothelin that mediate the immediate benefit of increased acid excretion, but their chronic upregulation promotes inflammation and fibrosis. It was also proposed that the new renal HCO_3_^−^ synthesis in response to metabolic acidosis leads to calcium precipitation in the kidney [21].

One of key findings in this study is that serum HCO_3_^−^ levels in some patients of lower HCO_3_^−^ group were within the normal range (22–25.3 mEq/L). At present, the Kidney Disease: Improving Global Outcomes (KDIGO) guidelines recommends to start the administration of alkali when serum HCO_3_^−^ levels are < 22 mEq/L, to maintain the value within the normal range [11]. On the other hand, previous study showed that H^+^ retention of variable severity has already occurred even if serum HCO_3_^−^ levels are within the normal range in patients with CKD, which is referred as subclinical metabolic acidosis [22–24]. Epidemiologic studies have showed that CKD patients with low-normal HCO_3_^−^ levels have higher risk of eGFR decline and mortality than those with high-normal HCO_3_^−^ levels [6, 25]. A randomized study in CKD patients with normal HCO_3_^−^ levels has also showed that sodium bicarbonate (NaHCO_3_) treatment preserved their kidney function after 10 years compared with sodium chloride (NaCl) administration or usual care [26]. Therefore, our result suggests the requirement of earlier intervention before overt metabolic acidosis develops, although both the precise prevalence of subclinical metabolic acidosis and optimal range for the correction of serum HCO_3_^−^ levels remain to be clarified. Further studies are needed to clarify these issues.

Another key finding in this study is that all-cause mortality was included in the composite outcomes we set. In accordance with our result, epidemiological studies have showed that higher HCO_3_^−^ levels within the normal range are associated with better survival [6, 25]. On the other hand, there is few data of the effect of alkali on the mortality in patients with CKD [27]. Because it was reported that metabolic acidosis can cause insulin resistance, skeletal muscle breakdown or cardiovascular disease as well as the progression of kidney diseases [7, 28, 29], our result that patients with lower HCO_3_^−^ levels had poorer prognosis might be reasonable. Further interventional studies are needed to identify the relationships between metabolic acidosis and mortality in patients with CKD.

Our study has several limitations. First, due to the retrospective study design, a longitudinal causal relationship cannot be established. However, to minimize this limitation, we employed the method of propensity score matching analyses in this study. Second, because of the relatively small number of patients in our cohort, the generalizability of our conclusions remains unclear.

## Conclusion

In conclusion, we showed here that CKD patients with lower HCO_3_^−^ levels, as compared with those with higher HCO_3_^−^ levels, were more likely to progress their kidney disease and have poorer prognosis. Therefore, metabolic acidosis may be a modifiable risk factor for the progression of CKD.

## Data Availability

The datasets used and/or analyzed during the current study are available from the corresponding author upon reasonable request.

## Abbreviations

ADPKD: Autosomal dominant polycystic kidney disease
Ca: Calcium
CKD: chronic kidney disease
CRP: C-reactive protein
CVD: Cardiovascular disease
eGFR: Estimated glomerular filtration rate
H^+^: Hydrogen ions
HCO_3_^−^: Bicarbonate
KDIGO: Kidney Disease: Improving Global Outcomes
LDL: Low-density lipoprotein
NaCl: Sodium chloride
NaHCO_3_: Sodium bicarbonate
Pi: Inorganic phosphorus
RAS: Renin-angiotensin system
SD: Standard deviation

## References

[1] Hamm LL, Nakhoul N, Hering-Smith KS (2015). Acid-Base Homeostasis. Clin J Am Soc Nephrol.

[2] Kraut JA, Madias NE (2016). Metabolic acidosis of CKD: an update. Am J Kidney Dis.

[3] Goraya N, Wesson DE (2017). Management of the Metabolic Acidosis of chronic kidney disease. Adv Chronic Kidney Dis.

[4] Yaqoob MM (2010). Acidosis and progression of chronic kidney disease. Curr Opin Nephrol Hypertens.

[5] Shah SN, Abramowitz M, Hostetter TH, Melamed ML (2009). Serum bicarbonate levels and the progression of kidney disease: a cohort study. Am J Kidney Dis.

[6] Raphael KL, Wei G, Baird BC, Greene T, Beddhu S (2011). Higher serum bicarbonate levels within the normal range are associated with better survival and renal outcomes in African Americans. Kidney Int.

[7] Dobre M, Yang W, Chen J, Drawz P, Hamm LL, Horwitz E (2013). Association of serum bicarbonate with risk of renal and cardiovascular outcomes in CKD: a report from the chronic renal insufficiency cohort (CRIC) study. Am J Kidney Dis.

[8] Jeong J, Kwon SK, Kim HY (2014). Effect of bicarbonate supplementation on renal function and nutritional indices in predialysis advanced chronic kidney disease. Electrolyte Blood Press.

[9] Goraya N, Simoni J, Jo CH, Wesson DE (2014). Treatment of metabolic acidosis in patients with stage 3 chronic kidney disease with fruits and vegetables or oral bicarbonate reduces urine angiotensinogen and preserves glomerular filtration rate. Kidney Int.

[10] Raphael KL, Greene T, Wei G, Bullshoe T, Tuttle K, Cheung AK (2020). Sodium bicarbonate supplementation and urinary TGF-beta1 in nonacidotic diabetic kidney disease: a randomized, controlled trial. Clin J Am Soc Nephrol.

[11] Improving Global Outcomes (KDIGO) CKD Work Group (2013). KDOGO 2012 clinical practice guideline for the evaluation and management of chronic kidney disease. Kiney Int Suppl.

[12] Wesson DE, Buysse JM, Bushinsky DA (2020). Mechanisms of metabolic acidosis-induced kidney injury in chronic kidney disease. J Am Soc Nephrol.

[13] Connors AF, Speroff T, Dawson NV, Thomas C, Harrell FE, Wagner D (1996). The effectiveness of right heart catheterization in the initial care of critically ill patients. SUPPORT Investigators JAMA.

[14] Uchida S, Chang WX, Ota T, Tamura Y, Shiraishi T, Kumagai T (2015). Targeting uric acid and the inhibition of progression to end-stage renal disease--a propensity score analysis. PLoS One.

[15] Phisitkul S, Hacker C, Simoni J, Tran RM, Wesson DE (2008). Dietary protein causes a decline in the glomerular filtration rate of the remnant kidney mediated by metabolic acidosis and endothelin receptors. Kidney Int.

[16] Wesson DE, Simoni J (2009). Increased tissue acid mediates a progressive decline in the glomerular filtration rate of animals with reduced nephron mass. Kidney Int.

[17] de Brito-Ashurst I, Varagunam M, Raftery MJ, Yaqoob MM (2009). Bicarbonate supplementation slows progression of CKD and improves nutritional status. J Am Soc Nephrol.

[18] Nath KA, Hostetter MK, Hostetter TH (1991). Increased ammoniagenesis as a determinant of progressive renal injury. Am J Kidney Dis.

[19] Wesson DE, Simoni J, Broglio K, Sheather S (2011). Acid retention accompanies reduced GFR in humans and increases plasma levels of endothelin and aldosterone. Am J Physiol Renal Physiol.

[20] Wesson DE, Jo CH, Simoni J (2015). Angiotensin II-mediated GFR decline in subtotal nephrectomy is due to acid retention associated with reduced GFR. Nephrol Dial Transplant.

[21] Halperin ML, Ethier JH, Kamel KS (1989). Ammonium excretion in chronic metabolic acidosis: benefits and risks. Am J Kidney Dis.

[22] Raphael KL (2018). Metabolic acidosis and subclinical metabolic acidosis in CKD. J Am Soc Nephrol.

[23] Goraya N, Simoni J, Sager LN, Mamun A, Madias NE, Wesson DE (2019). Urine citrate excretion identifies changes in acid retention as eGFR declines in patients with chronic kidney disease. Am J Physiol Renal Physiol..

[24] Goraya N, Simoni J, Sager LN, Madias NE, Wesson DE (2019). Urine citrate excretion as a marker of acid retention in patients with chronic kidney disease without overt metabolic acidosis. Kidney Int.

[25] Navaneethan SD, Schold JD, Arrigain S, Jolly SE, Wehbe E, Raina R (2011). Serum bicarbonate and mortality in stage 3 and stage 4 chronic kidney disease. Clin J Am Soc Nephrol.

[26] Mahajan A, Simoni J, Sheather SJ, Broglio KR, Rajab MH, Wesson DE (2010). Daily oral sodium bicarbonate preserves glomerular filtration rate by slowing its decline in early hypertensive nephropathy. Kidney Int.

[27] Morooka H, Yamamoto J, Tanaka A, Inaguma D, Maruyama S (2021). Relationship between mortality and use of sodium bicarbonate at the time of dialysis initiation: a prospective observational study. BMC Nephrol.

[28] Kobayashi S, Maesato K, Moriya H, Ohtake T, Ikeda T (2005). Insulin resistance in patients with chronic kidney disease. Am J Kidney Dis.

[29] Rajan V, Mitch WE (1782). Ubiquitin, proteasomes and proteolytic mechanisms activated by kidney disease. Biochim Biophys Acta.

